# Efficacy and safety of radiofrequency thermocoagulation target therapy for lumbar disc herniation: a systematic review

**DOI:** 10.3389/fsurg.2025.1722318

**Published:** 2025-12-02

**Authors:** TianTian Cui, WeiJie Chen, ChunXiang Lui, JiaLi An, WenKe Zheng

**Affiliations:** 1 Evidence-based Research Center, Tianjin University of Traditional Chinese Medicine, Tianjin, China; 2 Haihe Laboratory of Modern Chinese Medicine, Tianjin University of Traditional Chinese Medicine, Tianjin, China

**Keywords:** radiofrequency thermocoagulation target technique, lumbar disc herniation, efficacy, safety, meta-analysis

## Abstract

**Objective:**

To systematically evaluate the efficacy and safety of radiofrequency thermocoagulation target in the treatment of lumbar disc herniation.

**Methods:**

Clinical randomized controlled trials (RCTs) on RFTT for LDH were collected by searching databases including CNKI (China National Knowledge Infrastructure), Wanfang Data, VIP Database, PubMed, Cochrane Library, and Web of Science. The search period spanned from the establishment of each database to December 8, 2023. Two reviewers independently screened the literature and extracted data in accordance with the inclusion and exclusion criteria. The risk of bias of the included studies was assessed using the Cochrane Handbook 5.3.0 risk of bias assessment tool. Review Manager 5.4 software was used to analyze outcomes including total effective rate, Visual Analog Scale (VAS) score, Japanese Orthopaedic Association (JOA) score, Oswestry Disability Index (ODI), and safety indicators.

**Results:**

A total of 89 relevant studies involving 10, 079 participants were included, with 5,046 in the experimental group and 5,033 in the control group. The final analysis reviewed 89 studies, covering 10,079 participants. Among them, 5,046 participants were assigned to the experimental group and received the specific intervention, while the remaining 5,033 participants were in the control group and received conventional treatment. The analysis results showed that the total effective rate, JOA score, and ODI of the experimental group were significantly higher than those of the control group, indicating that the therapeutic effect of the experimental group was more pronounced than that of the control group. The incidence of adverse reactions was relatively consistent between the two groups, and most adverse reactions could be resolved through self-resolution without the need for intervention.

**Conclusion:**

In the treatment of LDH, radiofrequency thermocoagulation targeted surgery combined with conventional treatment is more effective than conventional treatment alone. It can better improve patients' quality of daily life and work ability, and this therapeutic approach significantly enhances patients' daily living quality and work capacity. However, due to the small sample size of the included studies and the low quality of relevant literature, the results of this study still require verification by more large-sample, multi-center, and high-quality clinical randomized controlled trials.

## 1 Introduction

Lumbar Disc Herniation (LDH) is a degenerative disease of the lumbar intervertebral disc that leads to rupture of the annulus fibrosus. Lumbar Disc Herniation (LDH) represents a degenerative condition affecting the lumbar intervertebral disc, resulting in the rupture of the annulus fibrosus. “A syndrome of low back pain, sciatica, lower limb numbness, lower limb weakness, indirect limp, and cauda equina syndrome, which is caused by protrusion of the nucleus pulposus and stimulation or compression of the nerve root and cauda equina nerve.” Clinically, according to the degree of protrusion and imaging characteristics, it is divided into bulging type, protruding type, prolapse type, and free type. According to surveys and studies, the global prevalence of LDH has reached 2%–4%, and it tends to be younger (1, 2, 94). With the aging of society in China and the increasing life pressure of young people, people's living habits have changed, leading to a significant increase in the incidence of lumbar disc protrusion. This disease commonly occurs in young and middle-aged people aged 25–55, and there are more male patients than female patients (3), which seriously affects people's quality of life.

At present, the treatment methods of lumbar disc herniation include surgical treatment and conservative treatment. Compared with surgical treatment, although conservative treatment does not cause obvious trauma and adverse reactions, it has a long treatment cycle and cannot relieve the symptoms of patients in a short time, and most patients cannot adhere to it. Currently, the treatment options for lumbar disc herniation encompass surgical and conservative approaches. While conservative treatment avoids significant trauma and adverse reactions compared to surgery, it features a prolonged treatment cycle and fails to promptly alleviate patients' symptoms, leading to poor adherence among many patients. Although conservative treatment can help patients relieve some discomfort, it is difficult to solve the fundamental problem (95). The continuous innovation of medical technology has made the minimally invasive interventional technology more and more mature. The target therapy of radiofrequency thermocoagulation has been used since March 2003. According to the follow-up observation of more than 10,000 patients for four years, more than 92% of lumbar disc herniation can be cured by this method. According to the follow-up observation of more than 10,000 patients for four years, more than 92% of lumbar disc herniation can be cured by this method. Studies have shown that radiofrequency thermocoagulation can effectively treat lumbar disc herniation, with success rates varying by method and patient group. In recent years, it has better advantages in the treatment of lumbar disc herniation because of its selectivity, specificity, less damage to the body and fewer complications (3, 4). Radiofrequency thermocoagulation is a minimally invasive surgery, it still belongs to the cRadiofrequency thermocoagulation, while considered minimally invasive, is still a form of surgery that can cause trauma. However, recent clinical studies have shown promising results regarding its efficacy and safety in treating lumbar disc herniation. For instance, one study reported that patients treated with radiofrequency thermocoagulation experienced significant improvement in symptoms and a high rate of patient satisfaction with minimal complications. Despite these positive findings, there remains a need for more high-quality clinical evidence to fully establish its effectiveness. Therefore, this article collected the randomized controlled trials of radiofrequency thermocoagulation in the treatment of lumbar disc herniation in detail, and used Meta-analysis to explore the efficacy and safety of radiofrequency thermocoagulation in the treatment of lumbar disc herniation, in order to provide reliable reference and evidence-based basis for the clinical application of radiofrequency thermocoagulation in the treatment of lumbar disc herniation.

## 2 Method

This review was carried out as per PRISMA guidelines. The systematic review protocol was not published.

### 2.1 Search strategy

Comprehensive databases such as CN-KI, WangFang, VIP, CBM(sinomed), Pubmed, Cochrane library, Web of Science, and EMBASE have systematically compiled randomized controlled trials (RCTs) that evaluate the efficacy of radiofrequency thermocoagulation in managing lumbar disc herniation. The search time limit was from the inception of each database to December 8, 2023, and the language was limited to Chinese/English. In addition, the references of the included literature were traced back to supplement the acquisition of relevant copy. Following the retrieval rules of each database, we adopted a method combining subject terms and free words. For the papers whose full texts could not be obtained, we searched the official websites of relevant journals and the Baidu Scholar website to retrieve randomized controlled trial (RCT) studies on radiofrequency ablation for lumbar disc herniation. In order to avoid literature omission, according to the “PICOS” principle (P(disease): Lumbar disc herniation; I (intervention): radiofrequency thermocoagulation target therapy; S (study type): RCT) was used to retrieve relevant literature. CNKI was taken as an example for Chinese database retrieval, and the search formula was as follows: (SU % = “randomized controlled trial” OR SU % = “randomized controlled trial” OR SU % = “clinical observation” OR SU % = “randomized controlled study”) AND (SU % = “Lumbar disc herniation” OR SU % = “Intervertebral disc herniation” OR SU % = “lumbar disc herniation” OR SU % = “lumbar disc herniation” OR SU % = “low back pain”) AND (SU % = “target radiofrequency thermocoagulation” OR SU % = “target radiofrequency thermocoagulation” OR SU % = “radiofrequency thermocoagulation treatment” OR SU % = “Radiofrequency thermocoagulation” OR SU % = “radiofrequency thermocoagulation”);The English database search takes Pubmed as an example, and the search formula is as follows:((((((((lumbar disk herniation) OR (lumbar intervertebral disc herniation)) OR (prolapse of lumbar intervertebral disc)) OR (lumbar disc herniation)) OR (lumbar disc protrusion)) OR (lumbar herniated disk)) OR (lumbar intervertebral disc prolapse)) AND ((((Radiofrequency thermocoagulation target technique) OR (radiofrequency thermocoagulation)) OR (radiofrequency heating-coagulated target)) OR (radiofrequency target))) AND ((((((rct) OR (rcts)) OR (randomized clinical trials)) OR (randomized controlled clinical trial)) OR (randomized controlled trials)) OR (randomized experiment)).

### 2.2 Inclusion and exclusion criteria

#### 2.2.1 Inclusion criteria

##### 2.2.1.1 Study types

Inclusion criteria encompassed all randomized controlled trials (RCTs) published in Chinese and English that evaluate treatments for lumbar disc herniation.

##### 2.2.1.2 Study subjects

All patients met the diagnostic criteria for lumbar disc herniation (LDH), regardless of their age or gender. Regardless of age or gender, all patients in this study met the established diagnostic criteria for lumbar disc herniation (LDH), which are consistent with the common clinical manifestations and imaging results described in medical literature. Referencing the current diagnostic criteria for lumbar disc herniation issued and implemented in China on October 13, 2012, and updated on December 10, 2021, the diagnostic criteria are as follows:

###### 2.2.1.2.1 Modern edicine

1. Diseases history: This disease is more common among young and middle-aged adults. In older people, it often recurs and is frequently associated with a history of lumbar trauma, cumulative injury or exposure to wind, cold and dampness.
2. Symptom: Low back pain, simple lumbar pain or radiating pain in the lower extremities. Limited lumbar movement, lateral bending, and increased pain when standing or walking.
3. Sign: Patients often present with abnormal changes in spinal posture, such as excessive lumbar lordosis, flattened or reversed lumbar lordosis, and lumbar scoliosis. Their spinal flexion, extension, lateral flexion, and rotation movements can all be restricted to varying degrees, with extension pain being the most prominent. Generally, there are obvious tender points at the intervertebral space of the affected spinous process and 1–2 cm beside the vertebrae, which often cause radiating pain in the lower extremities. A positive straight leg raising test and reinforcement test usually indicate intervertebral disc protrusion at L3-4, L4-5, or L5-S1, but a negative result does not rule out intervertebral disc protrusion above the L3-4 segment. A positive femoral nerve traction test usually indicates intervertebral disc protrusion at L2-3. The skin area innervated by the compressed nerve root will experience changes in sensation, initially hypersensitivity, followed by dullness or loss of sensation. Compression of the femoral nerve leads to a decreased patellar tendon reflex; compression of the S1 nerve root leads to a decreased Achilles tendon reflex. Some patients with a long course of disease and recurrent episodes often develop atrophy of the quadriceps femoris and calf muscles on the affected side.
4. x-rays examine technique: Routine anteroposterior and lateral lumbar spine radiographs are taken. The anteroposterior view shows vertebral rotation and scoliosis; the lateral view shows narrowed intervertebral spaces and reduced or even reversed lordosis. In middle-aged and elderly patients, intervertebral disc degeneration and osteophyte formation are often present. x-ray examination can also rule out bone and joint destruction, metastatic cancer, bone tuberculosis, tumors, and congenital spinal deformities.
5. CT examination: The direct image of the protrusion and its adjacent relationship with the nerve roots and dural sac can be observed, and the volume of the spinal canal, ligamentum flavum, nerve root canal and other conditions can also be understood. At the same time, the volume of the spinal canal and lateral recess can be measured from the cross-sectional images.
6. MRI examination: The size of intervertebral disc protrusion and the degree of compression on the dural sac and nerve roots can be determined.
7. Auxiliary examination: The main examination is electromyography, which can locate the damaged nerve roots. In some patients with a longer course of the disease, partial denervation signs may appear in the muscles innervated by the corresponding segments of the damaged nerves.

###### 2.2.1.2.2 TCM differentiation

1. Stagnation of blood: The patient experiences sharp pain in the waist and legs, which is fixed in location. The pain is milder during the day and more severe at night. It is difficult for the patient to bend or turn, and the pain intensifies when coughing. Occasionally, there is constipation and clear urine, along with irritability and dry mouth. The tongue is dark purple or has blood stasis spots, and the pulse is deep and sluggish.
2. Cold-dampness syndrome: Cold pain in the waist and spine, cold limbs with weakness, fixed pain upon palpation, sometimes numbness and heaviness in the lower extremities, severe pain when exposed to cold, pain alleviated by heat, long and clear urine. Pale tongue, thin white or greasy coating, deep and tight pulse.
3. Wind-dampness syndrome: Pain in the lumbar and dorsal regions radiates to the lower extremities, accompanied by numbness of the skin. The pain has no fixed location and wanders unpredictably, being related to changes in the weather. There is a slight aversion to wind and cold. The tongue is pale with a thin white or thin yellow coating, and the pulse is weak and fine.
4. Kidney-Yang deficiency: Persistent dull and aching pain in the lower back, cold and numbness in the limbs, weakness, and no improvement after long-term treatment. The patient prefers to be massaged and the symptoms worsen with exertion. Often accompanied by tightness and distension in the lower abdomen, pale complexion, aversion to cold, shortness of breath, and general weakness. The tongue is pale and the coating is thin and moist. The pulse is deep and weak.
5. Kidney-Yin deficiency: Persistent low back pain, weak and sore, not cured for a long time, aggravated by overwork. Often accompanied by restlessness, insomnia, dry mouth and throat, flushed face, and hot hands and feet. Red tongue with little coating, and taut, thin and rapid pulse (5).

##### 2.2.1.3 Intervention measures

The control group was the conventional treatment group of lumbar disc herniation, and the experimental group was treated with radiofrequency thermocoagulation target therapy on the basis of the conventional treatment of lumbar disc herniation.

##### 2.2.1.4 Main outcome measures

The modified Macnab criteria, Visual Analogue Scale (VAS) score, and Oswestry disability index (ODI) were used to evaluate disability, Japanese orthopedic association score (Japanese orthopedic association, JOA), serum inflammatory factor levels (serum IL-6, TNF-α, IL-1B), straight leg raising test, hemorheology indexes (thrombus length *in vitro* and wet weight *in vitro*), lower limb electromyography (electromyography F wave conduction of common peroneal nerve and posterior tibial nerve), recurrence rate, cure rate, etc.

##### 2.2.1.5 Adverse events

Nausea and vomiting, headache, limb numbness, postoperative lower limb pain aggravation, cauda equina nerve injury, postoperative infection, urinary retention, dizziness, psoas major muscle spasm, low back pain, etc.

#### 2.2.2 Exclusion criteria

1. Only one duplicate study was retained, and the articles that could not be extracted were excluded.
2. The experimental design lacked rigor, the original literature was incomplete, making it impossible to extract data from related papers or literatures featuring the same experimental data.

### 2.3 Literature quality evaluation and data extraction

#### 2.3.1 Assessor reliability evaluation

We used the Cochrane Collaboration's tool for assessing the risk of bias in randomized trials to evaluate the quality of randomized controlled trials. All studies were independently evaluated by two assessors. Before reaching a conclusion, any discrepant results between the assessors were discussed. If there were any inconsistencies in the results or opinions, a third party was introduced to adjudicate the differences.

And the Cohen's Kappa coefficient is utilized as a statistical metric for evaluating the consistency between two assessors in a classification task. It takes into account the difference between the agreements reached by the assessors and those that occur purely by chance. Compared with other measurement methods, it not only calculates the simple consistency between the model's predictions and the actual labels but also corrects for the consistency that may occur by chance. Therefore, it can provide a more accurate and reliable assessment result. Its formula is as follows:

$$\mathrm{Kappa}=\frac{\mathrm{Overall}\;\mathrm{accuracy}-\mathrm{Expected}\;\mathrm{accuracy}}{1-\mathrm{Expected}\;\mathrm{accuracy}}=\frac{N\sum_{i-1}^{n}\;X_{ii}-\sum_{i-1}^{n}\;(X_{i+}\times X_{-i})}{N^{2}-\sum_{i-1}^{n}\;(X_{i+}\times X_{-i})}$$

In the formula, *n* represents the category, N represents the total sum of the number of categories (referring to the number of inspection points in this context), $X_{\mathrm{ii}}$ represents the diagonal elements of the error matrix, $X_{i+}$ represents the total sum of the category column, $X_{-i}$ represents the total sum of rows by category.

Through this formula, the Kappa value can be obtained, ranging from −1 to 1:

A value of 1 indicates perfect agreement.
A value of 0 indicates that the degree of agreement is equivalent to that expected by chance.
Negative values indicate that the degree of agreement is less than that expected by chance.

The degree of agreement can be evaluated based on the value of the Kappa coefficient (Table 1):

**Table 1:** Kappa coefficient and degree of consistency.

Kappa coefficient	Degree of consistency
<0.4	Poor
0.4–0.6	Average
0.6–0.8	Good
>0.8	Excellent

##### 2.3.1.1 Operating steps

1. The initial literature set (*N* = 1,121) was labeled. A pseudo-random number sequence was generated using Python's “random()” function. Through a sorting algorithm, a representative sample of 500 articles was unbiasedly extracted.
2. Establish detailed, specific and operational assessment criteria: Ensure that all researchers have a clear and unified understanding of each aspect of the assessment to avoid subjective judgment differences caused by ambiguous standards. For detailed content, please refer to the inclusion and exclusion criteria.
3. Conduct unified training for evaluators: Ensure that all evaluators have received the same training, understand the purpose, standards, processes and methods of the assessment, to reduce subjective biases caused by differences in knowledge and experience.
4. Implement blind evaluation: During the assessment process, conceal the identity of the evaluated object or other background information that may affect the assessment results to prevent assessors from generating subjective biases due to preconceived notions or prejudices about the evaluated object.
5. Randomly assign samples: Randomly allocate the evaluation samples to different evaluators to ensure that each researcher's evaluated samples are representative and to avoid subjective judgment differences caused by sample selection bias. Two assessors independently completed the literature assessment in an environment free from interference. During the assessment process, the principle of blinding must be strictly followed, and information exchange is prohibited.
6. Conduct statistical analysis on the two evaluators, present the results in a table, and calculate the Kappa coefficient using the Scikit-learn package in Python.

##### 2.3.1.2 Interpretation of result

After analyzing the results of the two assessors, it was found that Assessor 1 selected 45 articles for inclusion and 455 for exclusion, while Assessor 2 selected 52 articles for inclusion and 448 for exclusion. The number of articles both assessors chose to include or exclude was 489, and the number of articles where one assessor chose to include and the other to exclude was 11. The Kappa value, rounded to three significant figures, *K* = 0.874. From this, it can be concluded that the consistency of literature screening is extremely high, with high transparency, low subjective bias and high credibility.

During the initial design phase of this study, we planned to use RoB 2. However, the study had a research cycle of 5 months, during which we needed to complete the quality assessment of 89 literatures. According to the RoB 2 assessment process, 28 specific questions in each domain must be answered for all included research literatures (cited from the RoB 2 Operation Manual), and the evaluation of a single literature took approximately 35 min. In contrast, the evaluation of a single literature using RoB 1 only took about 15 min. Furthermore, in practical operation, we found that RevMan 5.4, the systematic review management software commonly used by the team, only has a built-in RoB 1 assessment module. For RoB 2, external Excel toolkits or web templates are required (cited from the Cochrane RoB 2 Tool Instructions). Unfortunately, team members lacked experience in using this toolkit and had poor proficiency in its application. On the other hand, all team members had more than several years of experience in using RoB 1, which could significantly reduce operational errors. Therefore, to avoid misjudgment of the risk of bias caused by unskilled software operation and to balance “evaluation efficiency” and “result accuracy”, we finally chose RoB 1. In addition, we adopted the process of independent evaluation by two reviewers (Kappa = 0.874, indicating excellent consistency) to ensure the reliability of the risk of bias assessment results (Supplementary Appendix S4—The Cohen's Kappa coefficient). If more research resources are available in the future, we will attempt to conduct secondary verification on core literatures using RoB 2 to further verify the stability of the conclusions.

#### 2.3.2 Data extraction

##### 2.3.2.1 Preparatory phase

###### 2.3.2.1.1 Formulate the data extraction table

Based on the research question and inclusion criteria, determine the data content to be extracted and design the corresponding table. Common data extraction contents include study characteristics (such as author, title, publication year, study design, inclusion and exclusion criteria for research subjects, etc.), intervention measures (such as intervention methods, duration, etc.), outcome indicators (such as names of primary and secondary outcome indicators, time points, etc.) and data related to quality evaluation (such as randomization methods, etc.).

###### 2.3.2.1.2 Train data extraction personnel

All personnel involved in data extraction should receive unified training to familiarize themselves with the research purpose, inclusion and exclusion criteria, the usage method of the data extraction form, and the specific requirements for data extraction, etc., to ensure the accuracy and consistency of the extracted data.

##### 2.3.2.2. Data extraction

###### 2.3.2.2.1 Initial browse

Conduct a preliminary review of the included studies to understand their general content and structure, and determine the location and presentation format of the data in the articles, such as textual descriptions, tables, or charts.

###### 2.3.2.2.2 Detailed extraction

According to the content of the data extraction table, data should be extracted item by item from the included studies. For each piece of data, it is necessary to ensure that it is accurately obtained from the original text and filled in the corresponding position. During the extraction process, attention should be paid to the units, formats and precisions of the data to avoid misunderstandings or deviations.

##### 2.3.2.3 The verification and validation stage

###### 2.3.2.3.1 Double independent extraction

Data extraction is usually carried out independently by two researchers, and then their extraction results are compared to check for any discrepancies. If there are any differences, they need to discuss together and refer to the original text to determine the correct data.

###### 2.3.2.3.2 Cross-checking

In addition to double independent extraction, cross-checking can also be adopted. That is, after one researcher extracts the data, another researcher checks it, and vice versa, to further ensure the accuracy of the data.

##### 2.3.2.4 The sorting and filing stage

###### 2.3.2.4.1 Data organization

The extracted data were organized and summarized, classified and arranged according to research characteristics, intervention measures and outcome indicators, etc., to facilitate subsequent data analysis and synthesis.

###### 2.3.2.4.2 Data Archiving

Archive and preserve the extracted data along with the original literature, data extraction forms, etc., for easy reference and verification when needed. At the same time, record the problems encountered and the solutions adopted during the data extraction process to provide references for subsequent research.

Literature screening was performed by first reading the title, and after excluding obviously irrelevant literature, further reading the abstract and full text was performed to determine inclusion. The screening process began with reading the titles. Obviously irrelevant literature was excluded, followed by a detailed review of abstracts and full texts to determine inclusion. Original study authors were contacted by mail or telephone if necessary to obtain information that was not identified but was important to the study.

The data were extracted using a pre-developed data extraction form. The content of the extracted data included:

1. Basic information of the included studies: study title, first author, journal of publication, etc.
2. Baseline characteristics of participants and interventions.
3. Key elements of risk of bias assessment.
4. Outcome measures of interest and outcome measures.

Any study data that met the criteria were included in the final data synthesis. The above data has been sorted and saved in an Excel spreadsheet (Supplementary Table S1 in the Appendix).

#### 2.3.3 Literature risk of bias assessment

All studies were evaluated according to the Cochrane risk of bias tool criteria, including random sequence generation, allocation concealment, participant and personnel blinding, outcome assessor blinding, outcome data integrity (involving loss to follow-up), the possibility of selective reporting of study results, and the assessment of whether there were other sources of bias. The details are as follows:

##### 2.3.3.1 Random sequence generation (selection bias)

Carefully read all the included studies and examine the descriptions of the randomization methods in the studies, such as whether random number tables or computer-generated randomization software were used. Evaluate whether the randomization process for assigning subjects to different intervention groups is rigorous in the studies, and whether the randomization units (individuals, groups, etc.) and the time points of randomization are clearly indicated, to ensure that subjects are randomly assigned to each study group, thereby making the groups comparable in known and unknown prognostic factors and avoiding selection bias.

###### 2.3.3.1.1 Judgement Standard

Low risk of bias: Truly random methods were used, such as random number tables, computer-generated random numbers, etc., and the description was clear and detailed

High risk of bias: Non-random methods were used, such as allocation based on birth date, medical record number, etc., or although random methods were used, the description was vague and unclear, making it impossible to determine their randomness

Unclear: The randomization method was not clearly stated, or the provided information was insufficient to determine whether it was a true random method

##### 2.3.3.2 Allocation scheme concealment (selection bias)(selection bias)

Carefully read all the included studies and examine the descriptions in the studies regarding the concealment of the allocation scheme, such as whether effective concealment measures were taken, for example, using a central randomization system (such as telephone randomization or web-based randomization), sealed opaque envelopes, etc., to understand the generation and management of the allocation sequence, as well as the contact between researchers and participants during the randomization process, to ensure that the allocation scheme remains blinded to both researchers and participants before the subjects are assigned to specific intervention groups, and to avoid bias caused by premature leakage of allocation information.

###### 2.3.3.2.1 Judgement Standard

Low risk of bias: Appropriate allocation concealment methods were used, such as central randomization, sealed envelopes, etc., and were clearly described, ensuring that the allocation sequence was not known before randomization.

High risk of bias: No allocation concealment measures were taken, or the measures taken had obvious loopholes, such as using open randomization lists, predictable sequences, etc., which made it possible for the allocation results to be known in advance.

Unclear: Allocation concealment methods were not mentioned, or the description was ambiguous, making it impossible to determine whether effective allocation concealment measures were taken.

##### 2.3.3.3 Blind implementation (implementation bias and detection bias)

Carefully read all the included studies, and check the descriptions in the studies regarding the implementation of blinding to see if blinding was carried out for the study subjects, intervention implementers, and outcome assessors, as well as understand the specific methods and effects of blinding implementation, such as whether placebo controls were used and whether blinding was successfully maintained. For example, for subject blinding, it is necessary to consider whether it can effectively prevent subjects from changing their behavior or self-reporting of outcomes due to knowing their own group assignment; for assessor blinding, it is necessary to ensure that the measurers of outcome indicators do not know the group assignment of the subjects during the assessment process to ensure the objectivity of the measurement results.

###### 2.3.3.3.1 Judgement Standard

Low risk of bias: Blinding was successfully implemented for both the study participants, the intervention implementers, and the outcome assessors, and specific measures and effects of blinding were described, such as the use of placebo with identical appearance and the outcome assessors' unawareness of the group allocation.

High risk of bias: Blinding was not implemented, or it was implemented inadequately, such as blinding only a portion of the participants and potentially affecting the accuracy of the study results, or blinding was compromised without taking remedial measures.

Unclear: The implementation of blinding was not described in detail, or the provided information was insufficient to determine whether blinding was successfully implemented.

##### 2.3.3.4 Incomplete outcome data (attrition bias)

Carefully read all the included studies, and examine the descriptions of loss to follow-up and withdrawal in the studies, including the number of people lost to follow-up and withdrawn, the reasons, and whether they are related to the study outcomes. Determine whether there is a situation of missing outcome data in the studies. If there is missing data, did the researchers handle the missing data reasonably, such as conducting intention-to-treat analysis or making reasonable estimations of the missing data, to reduce the risk of attrition bias.

###### 2.3.3.4.1 Judgement Standard

Low risk of bias: The proportion of loss to follow-up and withdrawal is relatively low (e.g., less than 20%), and the methods for handling missing data are reasonable, such as conducting intention-to-treat analysis, or the reasons for loss to follow-up and withdrawal are not related to the study outcome.

High risk of bias: The proportion of loss to follow-up and withdrawal is relatively high (e.g., greater than 20%), and the methods for handling missing data are inappropriate, which may lead to bias in the study results, or the reasons for loss to follow-up and withdrawal are closely related to the study outcome and have not been handled reasonably.

Unclear: No detailed information on loss to follow-up and withdrawal is provided, or it is impossible to determine whether the missing data have affected the study results.

##### 2.3.3.5 Selective reporting (reporting bias)

Check whether the outcome indicators reported in the original research protocol (if available) of the study are consistent with those in the actual research report, and whether there is any selective reporting. This is to prevent researchers from selectively reporting results favorable to a certain intervention measure in the research report while ignoring or concealing other important but possibly unfavorable results for that intervention measure.

###### 2.3.3.5.1 Judgement Standard

Low risk of bias: All pre-specified outcome measures in the study protocol were fully reported, and detailed descriptions of the measurement methods and results for each outcome measure were provided.

High risk of bias: There were unreported outcome measures, and no reasonable explanations were given for the unreported outcome measures, or only outcome measures favorable to the research hypothesis were selectively reported.

Unclear: The study protocol or registration information could not be obtained, or it was not clear whether all pre-specified outcome measures were reported.

##### 2.3.3.6 Other biases

Carefully read the full text of the included studies and, based on the specific characteristics of the research and domain knowledge, determine whether there are other potential sources of bias. Examine the descriptions in the studies regarding other bias factors that may affect the accuracy and reliability of the research results beyond the aforementioned bias types, such as the influence of the research funder, conflicts of interest between the researchers and participants, and operational biases during the research implementation process. For instance, if a study is funded by a certain drug manufacturer, there may be a potential risk of bias where the researchers tend to draw conclusions favorable to the drug's efficacy of that manufacturer.

###### 2.3.3.6.1 Judgement Standard

Low risk of bias: No other potential sources of bias that could affect the study results were identified, or the identified other sources of bias had a minor impact on the study results.

High risk of bias: Obvious other sources of bias exist, such as the study funder possibly influencing the reporting and interpretation of the study results, or the researchers and participants having conflicts of interest that were not appropriately handled, etc.

Unclear: It is impossible to determine whether other sources of bias exist, or the extent of the impact of the identified other sources of bias cannot be accurately judged.

### 2.4 The risk of bias of included studies was evaluated

Two researchers independently evaluated the risk of bias of the included studies and cross-checked the results. The revised Cochrane Handbook5.3.0 was used to assess the risk of bias of the included literature. The risk of bias was assessed in seven domains using prespecified signaling questions as a guide:

1. Generation of random order.
2. The randomized scheme's allocation was concealed, and implementation bias was monitored.
3. Blinding and detection/measurement bias measures were applied to both participants and intervention implementors.
4. The outcome evaluation was conducted with blinding, and measures were taken to address lost-to-follow-up bias.
5. completeness of outcome index data (loss to follow-up) and reporting bias.
6. the possibility of selective reporting of research results and other biases.

Other sources of bias. For each domain, the risk of bias could be described as “low risk,” “some concern,” or “high risk.”

The overall risk of bias was judged for each study based on the risk of bias in each domain. The investigators performed and cross-checked, and when there was disagreement. Review Manager5.4 software was used to draw quality assessment charts.

### 2.5 Statistical methods

The Review Manager 5.4 software provided by the Cochrane Collaboration was used to analyze the statistical data of the included RCTs. Continuous data were expressed as mean difference (MD), and binary data were expressed as risk ratio (RR). All data analyses were conducted using 95% confidence intervals (CI). When *P* > 0.1 and <50%, heterogeneity was considered small, and a fixed-effect model was used; when *P* < 0.1 and >50%, heterogeneity was considered large, and a random-effect model was used. The sources of heterogeneity were discussed by excluding each study one by one. Sensitivity analysis was used to test the stability of the meta-analysis statistical results, and funnel plot analysis was used to describe potential publication bias. The Egger value of the data was calculated and the Egger publication bias plot was drawn using Stata 17 software. When the Egger value is greater than 0.05, it indicates that there is no obvious publication bias; otherwise, it indicates that there is a significant publication bias.

## 3 Result

### 3.1 Results of literature search

**Figure 1:**
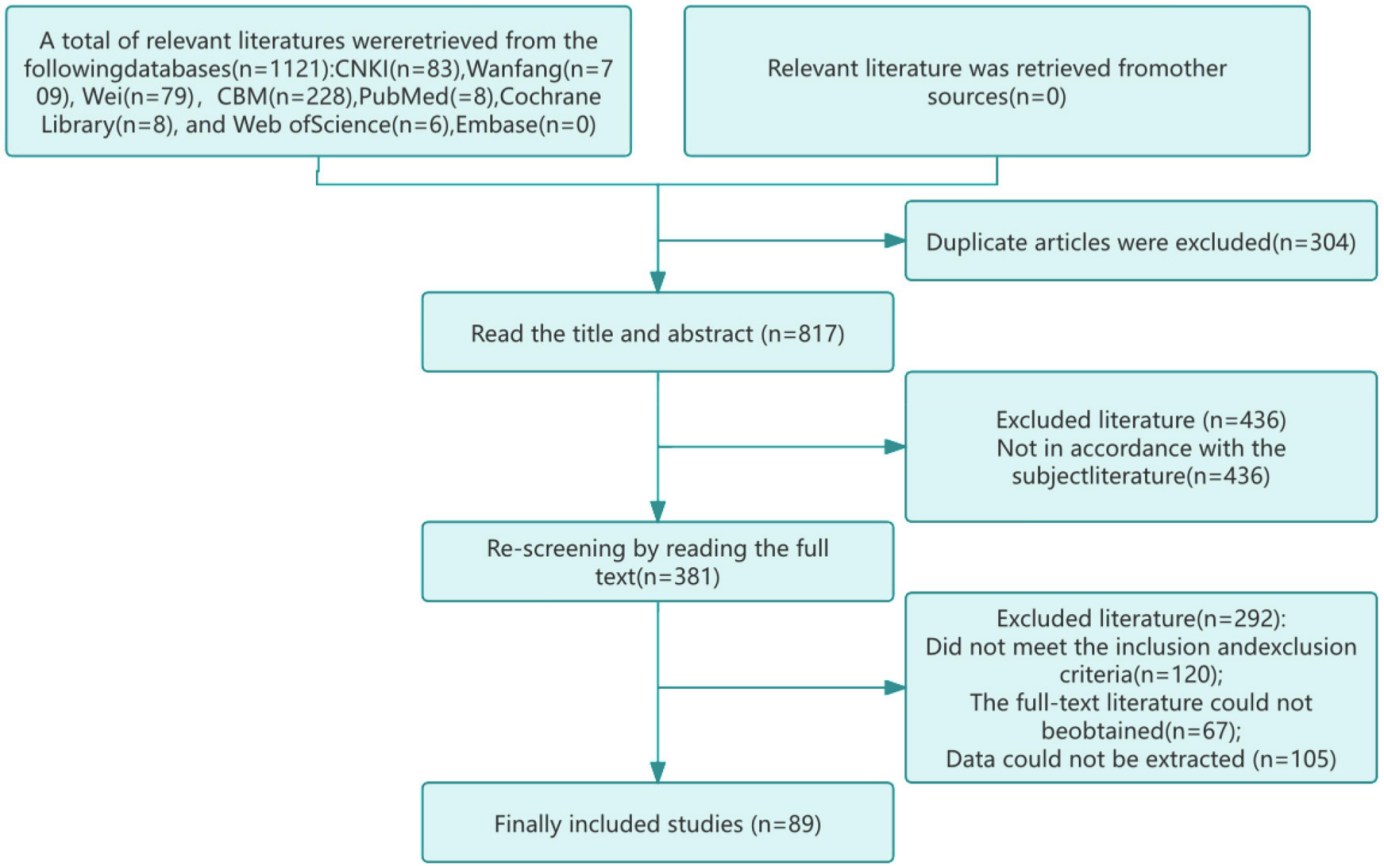
Literature search flow chart.

Initially, a comprehensive literature search was conducted using computerized databases and manual methods, retrieving a total of 1,121 articles. This included 83 articles from CNKI, 709 from WangFang, 79 from VIP, 228 from CBM(sinomed), 8 from Pubmed, 8 from the Cochrane library, 6 from Web of Science, and none from EMBASE. In the study, a total of 1,099 articles in Chinese and 22 in English were included, reflecting a comprehensive literature search and selection process. The literature was stored and managed using EndNote21 software, with initial duplicates being screened out. And read the title and abstract for further screening. Finally, read the full text and screen again. A total of 89 RCTS were included, including 88 in Chinese and 1 in English. The literature search and screening process is detailed in the flow chart presented in Figure 1, which outlines the stages of initial screening, secondary screening, and fine-grained selection.

### 3.2 Fundamental characteristics of included studies

A total of 89 articles were included in this study (6, 7), all of which were randomized controlled trials published from 2009 to 2023, involving 10, 079 patients. The interventions included in the study included 26 kinds of ozone ablation, selective nerve root block therapy, skin laser disc decompression (PLDD), small needle knife treatment, collagenase chemical dissolution therapy, oral Chinese medicine decoction, etc. The basic characteristics of the literature are detailed in Table 2.

**Table 2:** Basic characteristics.

Included studies	Sample size (T/C)	Age (years, $\bar{x}$ ± s)		Gender (number, male/female))		Interventions		Duration of disease (years)		Outcome indicator
		T	C	T	C	T	C	T	C	
Ming Lei (8)	75/75	40.3		78/72		GRT + Conventional therapy	Conventional therapy	0.25–13		②③
YuMing Chen (9)	45/45	46.2 ± 7.1	47.1 ± 6.7	25/20	26/19	GRT + Conventional therapyn	Conventional therapy	1.275 ± 0.5	1.317 ± 0.5	③
Bo Nian (6)	40/40	32		48/32		GRT + Conventional therapy	Conventional therapy	0.5–7		②
Li Wang (10)	75/75	40.8		88/62		GRT + Conventional therapy	Conventional therapy	0∼14		②③
FuWen Pan (11)	30/30	39		38/22		GRT + Conventional therapy	Conventional therapy	10		③
ChangCun Shao (12)	32/31	37.8 ± 6.2	38.5 ± 6.6	18/14	18/13	GRT + Conventional therapy	Conventional therapy	4.8 ± 1.6	4.2 ± 1.3	②③④⑤⑥
QiuAn Mei (13)	52/52	43.8 ± 10.8	42.9 ± 12.0	33/19	32/20	GRT + Conventional therapy	Conventional therapy	2.35 ± 0.54	2.37 ± 0.50	③④⑤⑥⑦⑩
HaiJun Liu (14)	66/66	46.2 ± 7.4	45.4 ± 7.1	38/28	42/24	GRT + Conventional therapy	Conventional therapy	28.6 ± 6.7	29.2 ± 6.8	②③④
HengChao Luo (15)	58/58	35.85 ± 3.64	35.79 ± 3.72	45/13	48/10	GRT + Conventional therapy	Conventional therapy	7.08 ± 2.11	7.13 ± 2.09	⑤⑥
JingYi Wei (16)	44/44	44.05 ± 4.92	42.15 ± 4.87	25/19	23/21	GRT + Conventional therapy	Conventional therapy	4.67 ± 0.81	4.61 ± 0.79	②③④⑤
JinYi Huang (17)	50/50	67.25 ± 3.72	65.74 ± 3.77	21/29	23/27	GRT + Conventional therapy	Conventional therapy	4.53 ± 0.85	4.37 ± 0.84	②③④⑤⑥
WenYi Gu (18)	35/35	44.68 ± 4.26	44.68 ± 4.26	15/20	13/22	GRT + Conventional therapy	Conventional therapy	Not mentioned		⑩
GuanLin Lin (19)	42/42	52.69 ± 2.53	52.61 ± 2.48	26/16	26/16	GRT + Conventional therapy	Conventional therapy	1.0325 ± 0.1383	1.0283 ± 0.135	⑩⑥
Qing Zhang (20)	35/35	35.71 ± 3.46	35.68 ± 3.44	28/7	29/6	GRT + Conventional therapy	Conventional therapy	6.73 ± 2.04	6.71 ± 2.01	②③⑤⑩
WenFU Zhang (21)	35/35	36.2 ± 3.72	36.2 ± 3.72	27/8	28/7	GRT + Conventional therapy	Conventional therapy	6.73 ± 2.09	6.77 ± 2.04	②
XiJuan Wu (22)	40/40	50.24 ± 3.17		42/38		GRT + Conventional therapy	GRT	4.76 ± 1.33		⑩③
JingBin Wu (23)	40/40	75.41 ± 2.99	75.23 ± 2.52	23/17	24/16	GRT + Conventional therapy	Conventional therapy	2.26 ± 0.72	2.24 ± 0.75	②④
RenHuan Wang (24)	50/50	49.4 ± 3.6	49.2 ± 3.5	22/28	21/29	GRT + Conventional therapy	Conventional therapy	2.7 ± 0.8	2.6 ± 0.7	①③
GuoFu Li (25)	20/20	46.23 ± 5.42	45.92 ± 5.34	8/12	9/11	GRT + Conventional therapy	Conventional therapy	Not mentioned		②③⑤
ZongJun Quan (26)	20/20	54.19 ± 7.28	52.47 ± 6.25	12/8	11/9	GRT + Conventional therapy	Conventional therapy	Not mentioned		②④⑤
LiLi Wu (27)	21/21	51.4 ± 6.1	50.6 ± 5.9	12/9	13/8	GRT + Conventional therapy	Conventional therapy	3.4 ± 2.6	3.2 ± 2.1	②③
Lei Feng (28)	33/33	46.2 ± 7.3	45.3 ± 7.1	19/14	21/12	GRT + Conventional therapy	Conventional therapy	2.358 ± 0.508	2.425 ± 0.558	②③④
HongGuang Yu (29)	40/40	39.25 ± 4.07	40.18 ± 4.37	21/11	29/19	GRT + Conventional therapy	Conventional therapy	Not mentioned		③④⑤
Zun Wei (30)	46/46	45.83 ± 5.92	44.97 ± 5.74	23/23	24/22	GRT + Conventional therapy	Conventional therapy	3.96 ± 1.52	3.76 ± 1.43	①③⑤⑩
Bo Zhao (31)	53/53	32.2 ± 3.2	34.1 ± 3.1	30/23	33/20	GRT + Conventional therapy	Conventional therapy	10.8 ± 5.1	11.4 ± 5.3	③⑦
LiQun Du (32)	64/64	42.39 ± 7.28	43.21 ± 7.54	39/25	37/27	GRT + Conventional therapy	Conventional therapy	2.17 ± 0.95	2.48 ± 1.24	③④⑤⑩
ChengZhe Cao (33)	105/105	41.25 ± 3.74	39.68 ± 3.24	59/46	52/53	GRT + Conventional therapy	Conventional therapy	0.56 ± 0.2	0.58 ± 0.209	③⑤⑧
LiJun Cui (34)	30/30	53.89 ± 4.61	54.21 ± 4.90	16/14	15/15	GRT + Conventional therapy	Conventional therapy	0.558 ± 0.276	1.316 ± 0.264	②③
XingFu Jiang (35)	57/56	59.12 ± 6.53	58.34 ± 6.71	33/24	31/25	GRT + Conventional therapy	Conventional therapy	1.47 ± 0.32	1.43 ± 0.30	⑥⑩
ChaoLin Yu (36)	41/41	57.36 ± 3.39	57.92 ± 3.23	25/16	24/17	GRT + Conventional therapy	Conventional therapy	9.44 ± 1.52	9.03 ± 1.61	②③
Xin Xu (37)	43/43	47.6 ± 10.9	46.2 ± 10.4	27/16	28/15	GRT + Conventional therapy	Conventional therapy	Not mentioned		①②③
ZhenGang Zhai (38)	50/50	54.6 ± 4.7	54.4 ± 4.6	23/27	22/28	GRT + Conventional therapy	Conventional therapy	Not mentioned		②⑩
HaiBao Tian (39)	46/46	52.84 ± 7.43	52.94 ± 7.62	25/21	26/20	GRT + Conventional therapy	Conventional therapy	4.30 ± 2.94	4.25 ± 2.38	②③
YanDe Wang (40)	41/41	43.11 ± 5.13	43.14 ± 5.11	25/16	24/17	GRT + Conventional therapy	Conventional therapy	3.89 ± 1.83	3.84 ± 1.88	②③
BoLong Lin (41)	42/42	36.3 ± 8.7	37.4 ± 9.2	23/19	25/17	GRT + Conventional therapy	Conventional therapy	0.575 ± 0.067	0.6 ± 0.108	①③⑤⑧⑩
PaiZhou Zhu (42)	45/45	40.6 ± 8.5	40.5 ± 8.6	28/17	30/15	GRT + Conventional therapy	Conventional therapy	1.73 ± 0.43	1.708 ± 0.44	②③④
WeiGuo Zhang (43)	45/45	55.2 ± 12.6	56.4 ± 11.4	27/17	29/16	GRT + Conventional therapy	Conventional therapy	5.4 ± 2.2	5.7 ± 2.6	③⑥
YunQiang Cui (44)	45/45	46.32 ± 8.46	45.79 ± 8.89	24/21	23/22	GRT + Conventional therapy	Conventional therapy	2.34 ± 0.469	2.302 ± 0.44	⑤⑥⑩
Zheng Lei (45)	60/60	50.4 ± 12.9	52.3 ± 13.2	38/22	40/20	GRT + Conventional therapy	Conventional therapy	4.5 ± 2.9	4.2 ± 2.6	②④
QiuAn Mei (46)	32/32	50.03 ± 4.28	48.67 ± 7.31	14/18	12/20	GRT + Conventional therapy	Conventional therapy	2.38 ± 0.23	2.58 ± 0.11	③⑤⑩
YuBing Liang (47)	463/463	49.7 ± 13.5	52.6 ± 11.3	275/188	267/196	GRT + Conventional therapy	Conventional therapy	0.08∼5	0.08∼5.5	③⑩
XueZhong Du (48)	32/32	43.39 ± 5.27	42.77 ± 4.92	14/18	17/15	GRT + Conventional therapy	Conventional therapy	0.718 ± 0.212	0.75 ± 0.194	②③⑤
HuiChao Cao (49)	50/50	46.97 ± 3.76	47.23 ± 3.65	28/22	27/23	GRT + Conventional therapy	Conventional therapy	4.12 ± 2.24	4.23 ± 2.38	③⑨⑩
Qi Sun (50)	110/110	54.98 ± 1.2	52.80 ± 1.6	55/55	64/46	GRT + Conventional therapy	Conventional therapy	0.525 ± 0.1	0.408 ± 0.117	③⑩
ZunZhang Lui (51)	60/60	48.42 ± 8.35	47.28 ± 7.85	37/23	35/25	GRT + Conventional therapy	Conventional therapy	Not mentioned		②③⑩
LongTao Ren (52)	28/32	57.2 ± 5.1	57.4 ± 5.4	19/9	21/11	GRT + Conventional therapy	Conventional therapy	3.3 ± 50.3	3.4 ± 50.7	①⑩
FuSheng Han (53)	104/104	54 ± 13	53 ± 13	61/43	60/44	GRT + Conventional therapy	Conventional therapy	Not mentioned		③⑩
ZhongHua Yang (54)	30/30	47.07 ± 5.58	48.25 ± 6.32	18/12	5/15	GRT + Conventional therapy	Conventional therapy	Not mentioned		②③
LiFei Yu (55)	50/50	40 ± 1.3	42 ± 1.6	29/21	26/24	GRT + Conventional therapy	Conventional therapy	4∼11	3∼12	②
Jun Tan (56)	45/45	56.3 ± 3.8	53.3 ± 3.5	31/14	27/18	GRT + Conventional therapy	Conventional therapy	Not mentioned		③④⑤
ShuDong Dong (57)	88/88	36.35 ± 1.64	37.32 ± 1.56	45/43	46/42	GRT + Conventional therapy	Conventional therapy	5.26 ± 1.37	5.33 ± 1.52	⑩
ShaoZhong Lin (58)	30/30	43.6 ± 19.2		41/19		GRT + Conventional therapy	Conventional therapy	3.1 ± 5.5		②③
JunSong Zhu (59)	90/90	46.16 ± 11.72	42.2 ± 10.38	56/34	61/29	GRT + Conventional therapy	Conventional therapy	0.383 ± 0.23	0.283 ± 0.174	②⑥
HongJian Zhang (60)	44/44	Not mentioned		24/20	25/19	GRT + Conventional therapy	Conventional therapy	2.615 ± 0.38	2.621 ± 0.385	③⑦
XiaoFeng He (61)	53/53	40.2 ± 6.4	41.7 ± 5.9	25/28	27/26	GRT + Conventional therapy	Conventional therapy	3.4 ± 1.6	3.6 ± 2.3	③
FuChao He (62)	100/100	48.3 ± 0.3	48.3 ± 0.3	62/38	58/42	GRT + Conventional therapy	Conventional therapy	4.3 ± 1.3	4.1 ± 1.1	②③
JieYun Huang (63)	33/33	38.2 ± 2.6	39.5 ± 2.4	18/15	19/14	GRT + Conventional therapy	Conventional therapy	3.7 ± 0.8	3.5 ± 0.5	③
Yi Guo (64)	40/40	46.93 ± 9.08	46.2 ± 8.12	24/16	23/17	GRT + Conventional therapy	Conventional therapy	2.33 ± 1.0	2.49 ± 2.5	②③
ZhiFang Fan (65)	52/52	45.69 ± 1.74	43.69 ± 1.20	33/19	32/20	GRT + Conventional therapy	Conventional therapy	2.74 ± 0.52	2.15 ± 0.58	③⑩
ZhengLing Yang (66)	40/40	46.3 ± 8.2	46.5 ± 7.4	27/13	26/14	GRT + Conventional therapy	Conventional therapy	2.508 ± 0.617	2.391 ± 0.625	②③⑩
YanBo Yu (67)	30/25	17.5 ± 2.2	15.6 ± 1.9	17/13	15/10	GRT + Conventional therapy	Conventional therapy	Not mentioned		②③
GuoHui Zhou (68)	30/30	46.85 ± 10.25		51/39		GRT + Ozone injection	GRT	4.25 ± 2.58		③⑩
JunFang Wu (69)	44/44	50.31 ± 2.65	49.23 ± 2.54	21/23	20/24	GRT + Conventional therapy	Conventional therapy	2.51 ± 0.82	2.13 ± 0.54	①
Tao Liu (70)	68/67	50.7 ± 13.0	49.9 ± 13.1	41/27	42/25	GRT + Conventional therapy	Conventional therapy	4.1 ± 2.8	3.9 ± 2.7	②③④
FuChao He (71)	100/100	52.2 ± 5.3		114/86		GRT + Conventional therapy	Conventional therapy	Not mentioned		⑥
FuChao He (72)	100/100	52.1 ± 5.6	52.3 ± 5.7	57/43	56/44	GRT + Conventional therapy	Conventional therapy	Not mentioned		①
HaiBo Jiang (73)	50/50	50.2 ± 4.2	49.6 ± 3.7	26/24	28/22	GRT + Conventional therapy	Conventional therapy	1.525 ± 0.758	1.3 ± 0.725	③④
Lin Li (74)	36/36	30.2 ± 2.6	31.6 ± 2.7	24/12	25/11	GRT + Conventional therapy	Conventional therapy	Not mentioned		②⑨
ZhaoBao Li (75)	25/25	52.5	50.5	10/15	12/13	GRT + Conventional therapy	Conventional therapy	3.5	3	②③⑨
YuMing Chen (9)	45/45	46.2 ± 7.1	47.1 ± 6.7	25/20	26/19	GRT + Conventional therapy	Conventional therapy	1.275 ± 0.542	1.317 ± 0.517	③
Tao Guo (76)	40/40	30.35 ± 5.526	31.00 ± 5.12	28/12	22/18	GRT + Conventional therapy	Conventional therapy	1.396 ± 0.916	1.396 ± 0.764	②⑨
JinShan Zhang (77)	37/37	46.84 ± 5.52	47.16 ± 4.39	23/14	25/12	GRT + Conventional therapy	Conventional therapy	Not mentioned		③
Jie Zhang (78)	50/50	47 ± 4.88	46 ± 4.12	26/24	30/20	GRT + Conventional therapy	Conventional therapy	Not mentioned		⑩
Di Gao (79)	40/40	54.8 ± 11.4	55.2 ± 10.7	26/14	27/13	GRT + Conventional therapy	Conventional therapy	11.5 ± 4.7	10.9 ± 5.0	②③
LiLi Han (80)	48/54	44.9 ± 13.3	45.3 ± 12.1	28/12	30/24	GRT + Conventional therapy	Conventional therapy	2.975 ± 1.05	3.042 ± 1.175	②③
XiaoLin Yang (81)	16/16	46		20/12		GRT + Conventional therapy	Ozone ablation	Not mentioned		②③
KeMei Wu (82)	60/60	42.52 ± 5.23	43.17 ± 5.03	35/25	39/21	GRT + Conventional therapy	Conventional therapy	3.21 ± 0.18	3.19 ± 0.16	②
Tao Fan (83)	110/110	52.41 ± 8.15	51.38 ± 8.68	74/36	68/42	GRT + Conventional therapy	Conventional therapy	5.99 ± 4.84	6.44 ± 5.07	②③
Tao Cheng (84)	60/60	23∼68	19∼66	33/27	29/31	GRT + Conventional therapy	Conventional therapy	0.167∼0.5		③⑩
Xin Wang (85)	51/51	48.52 ± 9.61		89/64		GRT + Conventional therapy	Conventional therapy	3.51 ± 2.61		②③
Li Yang (86)	100/100	42.5 ± 8.9	44.1 ± 9.1	57/43	61/39	GRT + Conventional therapy	Conventional therapy	3.292 ± 1.075	3.208 ± 0.85	③⑤
YongKai Zhao (87)	168/152	45.3 ± 6.6	45.2 ± 6.5	98/70	92/60	GRT + Conventional therapy	Conventional therapy	1.4 ± 0.7	1.375 ± 0.683	③⑩
YuJie Sun (88)	80/80	43.7 ± 8.1		98/62		GRT + Conventional therapy	Conventional therapy	1.608 ± 0.575		②③⑤
JinChun Shan (89)	53/51	45.5	46.3	32/21	29/22	GRT + Conventional therapy	Conventional therapy	8.3	7.8	⑩
BoHua Li (90)	45/45	43.5 ± 3.7	42.7 ± 3.4	31/14	30/15	GRT + Conventional therapy	Conventional therapy	3.7 ± 2.0	3.9 ± 2.3	②③
JianPing Wu (91)	50/50	47.8 ± 3.2	49.0 ± 3.1	28/22	30/20	GRT + Conventional therapy	Conventional therapy	4.2 ± 2.7	4.0 ± 2.3	②③
ZhenYue Wang (92)	40/40	Not mentioned		24/16	22/18	GRT + Conventional therapy	Conventional therapy	2.577 ± 0.372	2.577 ± 0.372	③⑦
DaChun Li (7)	46/46	42.02 ± 8.19	43.15 ± 10.30	27/19	26/20	GRT + Conventional therapy	Conventional therapy	5.11 ± 1.09	4.92 ± 0.97	②
Sujeet Gautam (93)	46/45	45.11 ± 9.4	43.51 ± 9.9	25/18	27/14	GRT + Conventional therapy	Conventional therapy	Not mentioned		③④⑩

GRT, radiofrequency thermocoagulation target therapy; T, experimental group; C, control group. ① Total effective rate; ② Modified Macnab criteria; ③ VAS, Visual analogue scale; ④ ODI, Oswestry disability index; ⑤ JOA, Japanese orthopedic association score; ⑥ Serum inflammatory factor levels (serum IL-6, TNF-α, IL-1B), ⑦ straight leg raising test, ⑧ hemorheology indexes (thrombus length and wet weight *in vitro*), ⑨ lower limb electromyography (F wave conduction of common peroneal nerve and posterior tibial nerve electromyography), ⑩ clinical efficacy. Conventional therapy:Ozone injection, Qiangyao-huoxue soup, Tongbi decoction, Taohongsiwu decoction, Traditional Chinese Medical method, Duhuo Jisheng Decoction, Percutaneous laser disc decompression, A small dose of collagenase, Release with small needle knife, Acupotomy, Chinese medicine, acupuncture and rehabilitation, Acupuncture triple therapy Bushen Zhuanggu decoction, Bushen Zhuanggu prescription, Ablation of the nucleus pulposus, ect.

### 3.3 The quality and risk of bias of the included literature were evaluated

69 studies (6, 7, 13, 14, 16, 23, 27, 38, 39, 43, 45, 48–50, 53, 56–59, 68, 75, 76, 79, 82, 88, 91, 93) were randomized assignments using random sequence generation methods and 20 studies (15, 18, 20, 25, 29, 33, 35, 37, 47, 52, 61, 65, 69, 70, 73, 74, 77, 80, 89) were randomized assignments using non-random sequence generation methods.

The 15 studies (23, 28, 34, 50, 59, 68, 82, 88, 93) were marked as low risk of bias because the relevant contents mentioned by the authors made it impossible for researchers and research subjects to foresee the order of allocation during the selection and grouping process;21 studies (15, 18, 20, 29, 33, 35, 37, 47, 51, 52, 61, 65, 69, 70, 73, 77, 80, 89, 91) were marked as high risk of bias because the relevant contents mentioned by the authors allowed the researchers or research subjects to foresee the allocation sequence;53 studies (6–8, 13, 16, 17, 21, 27, 38, 39, 42, 43, 45, 49, 53, 56–58, 76, 79, 90) did not mention concealment of allocation schemes, so labelling was unclear.

In the 3 studies (70, 71, 89), the authors tried to blind the research subjects and researchers, but it was easy to be identified, and the results would be easily affected, so it was marked as high risk of bias;85 studies (6–8, 14, 16, 23, 29, 72, 88, 91) did not mention the blinding of outcome assessors, but the included indicators did not affect the final results, so they were marked as low risk of bias; 1 study (93), the authors' information about blinding was not sufficiently described, so the evaluators could not determine whether blinding was actually performed on the study subjects and investigators, so it was marked as unclear.

In 1 study (93), the authors did not mention the blinding method, so the evaluators could not judge whether the study really blinded the outcome evaluators, so it was marked as unclear; In 88 studies (6–8, 29), the authors mentioned that there was no blinding of outcome assessors, but it did not affect outcome assessment, so it was marked as low risk of bias.

In 3 studies (14, 70, 89), The reports lacked sufficient description of loss to follow-up and withdrawal information, or failed to mention outcome indicators in the methods section, leading to them being marked as unclear; the reasons for loss to follow-up in 86 studies (6, 7, 16, 51, 88, 91) were not closely related to the outcome indicators, which was not enough to have a clinically meaningful impact on the intervention effect, so it was marked as low risk of bias.

The relevant indicators of concern in the systematic review mentioned by the authors in 1 study (35) were not fully reported in that study and could not be included in the meta-analysis, so they were marked as high risk of bias. In 21 studies (7–9, 18, 22, 24, 38, 41, 55–57, 63, 74–76, 80, 81, 83, 92), the relevant data mentioned by the authors were not enough for the evaluators to judge, so they were marked as unclear. Among the 67 studies (6, 11, 12, 15, 17, 19, 20, 25, 26, 30–32, 37, 40, 45, 46, 48–50, 53, 54, 64, 66–68, 73, 79, 82, 85–87, 93), the authors did not report all the outcome measures of interest in the systematic review, but the study reported all the outcome measures listed in the methods, and the other unreported indicators were not available in the study protocol, so they were marked as low risk of bias.

**Figure 2:**
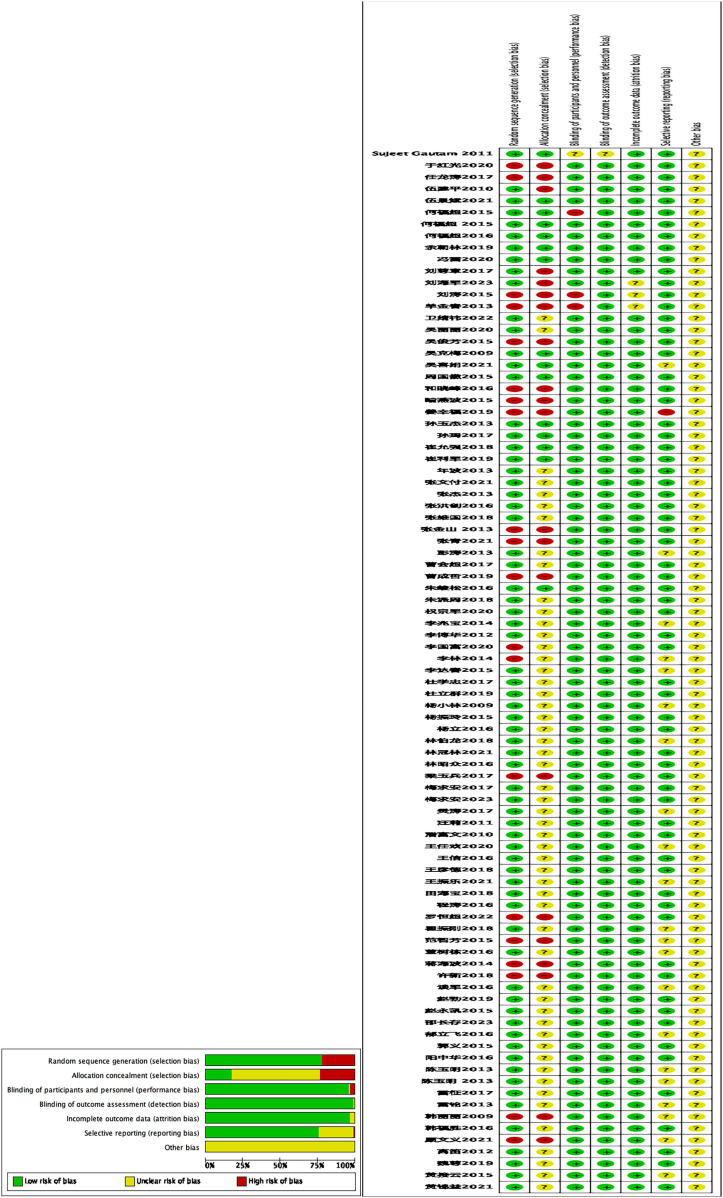
The is the bias risk assessment chart of the included studies.

No other bias was mentioned in any of the 89 studies. The detailed results of the quality and risk of bias assessment of the included literature are shown in Figure 2 and Supplementary Figure S1.

## 4 Results of meta-analysis

### 4.1 Total effective rate

**Figure 3:**
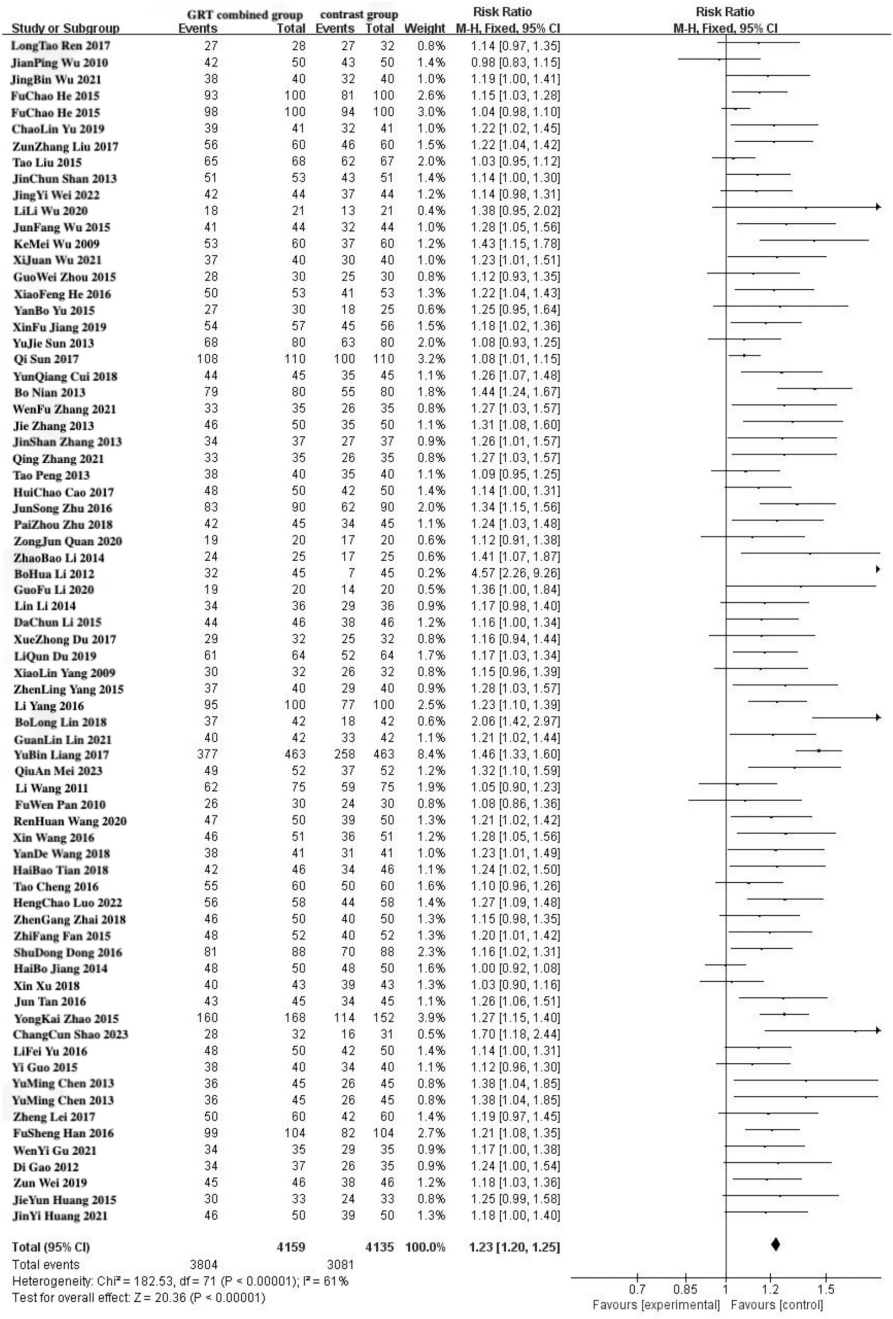
Forest plot of total effective rate comparison of the included studies.

**Figure 4:**
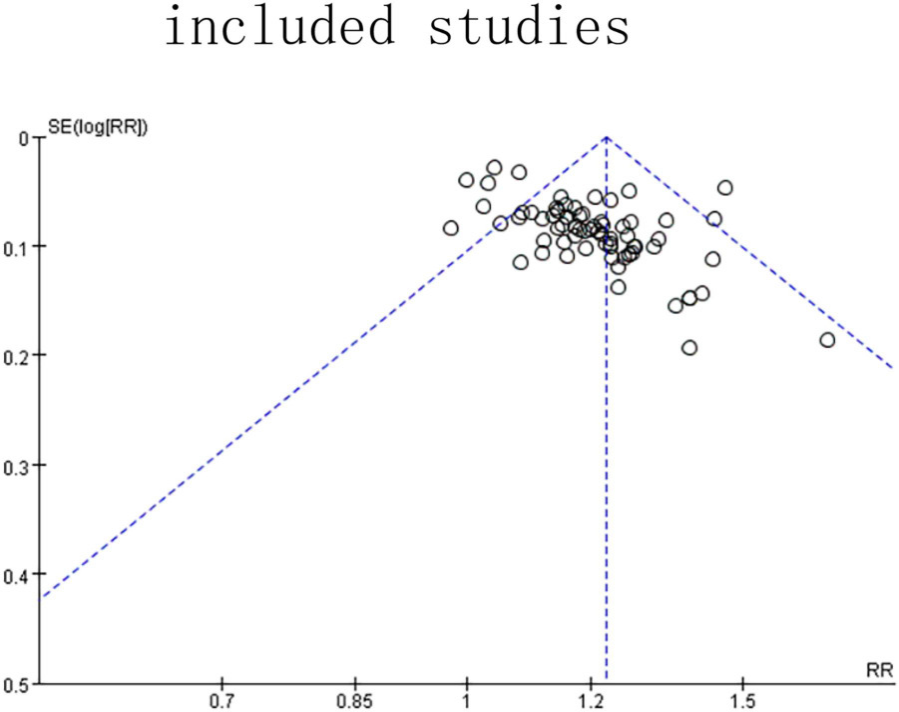
Funnel plot of the total response rate of the included studies.

**Figure 5:**
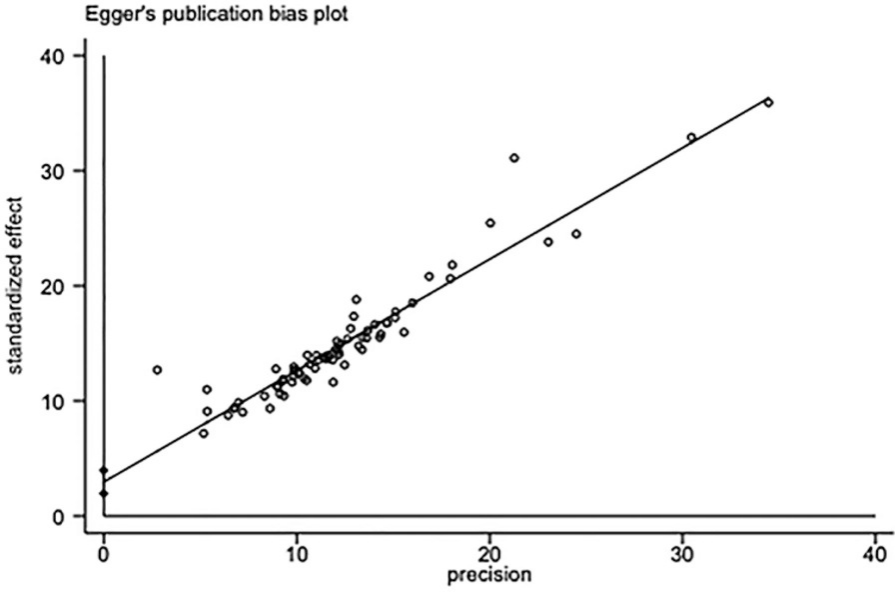
Egger's publication bias plot.

**Figure 6:**
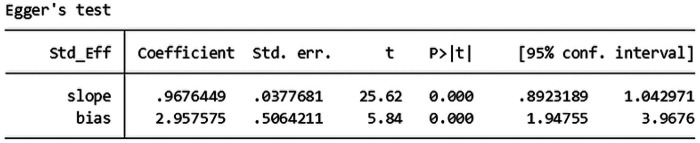
Egger's publication bias table.

**Figure 7:**
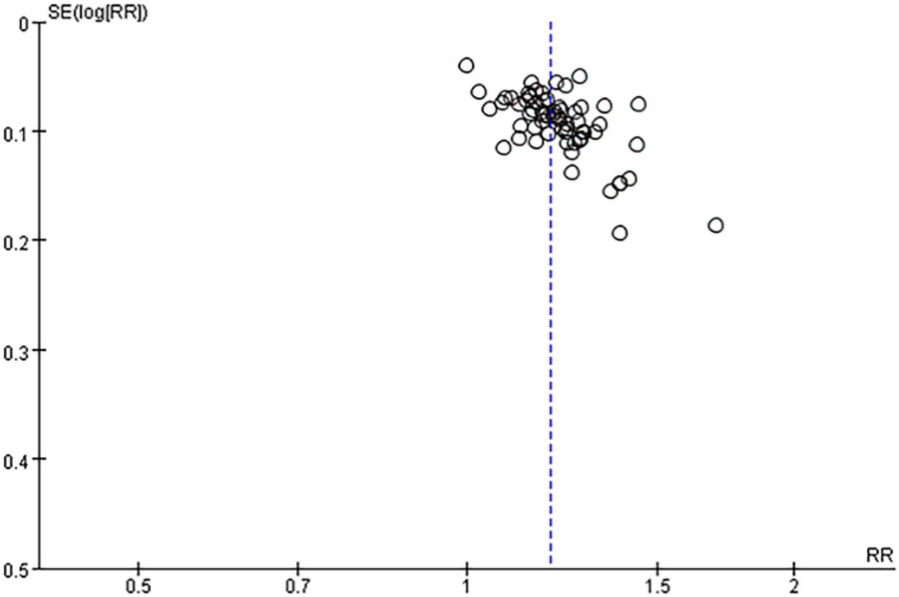
Funnel plot for comparison of total response rates of the included studies.

The total effective rate was used as the primary outcome index, and 72 studies (6, 7, 9, 12, 13, 19, 21, 24, 36, 44, 45, 47, 49, 51–53, 56, 59, 62, 64, 70, 71, 77, 78, 85–87) reported the total effective rate after treatment. A total of 8,294 patients were included in the study, with 4,159 patients in the experimental group and 4,135 patients in the control group. In the analysis of conventional treatment, the included studies were evaluated using the fixed effect model, as depicted in Figures 3, 4, with a significant result of *P* < 0.00001 and an I-squared statistic of 61%, suggesting that that Given the significant statistical heterogeneity across the studies, further sensitivity analysis was conducted on 72 studies, with detailed results presented in Section 2.1.1. Sensitivity analysis. For detailed data, see the forest plot of total effective rate comparison of the included studies in Figure 3 and the funnel plot of total effective rate comparison of the included studies in Figure 4. Egger's *P* < 0.0001, indicating a significant publication bias.(Figure 5. Egger's publication bias plot, Figure 6 Egger's publication bias table and Figure 7).

#### 4.1.1 Sensitivity analysis

**Figure 8:**
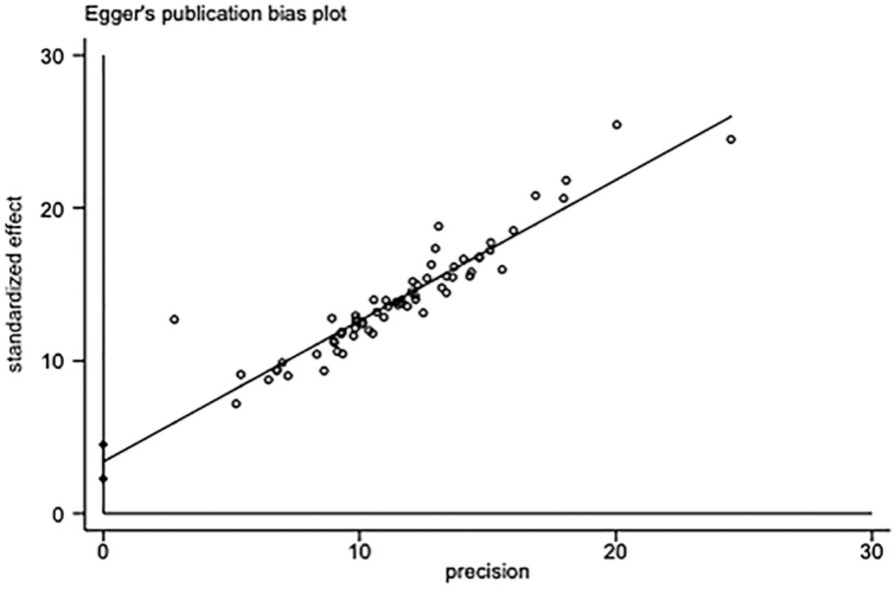
Egger's publication bias plot.

**Figure 9:**
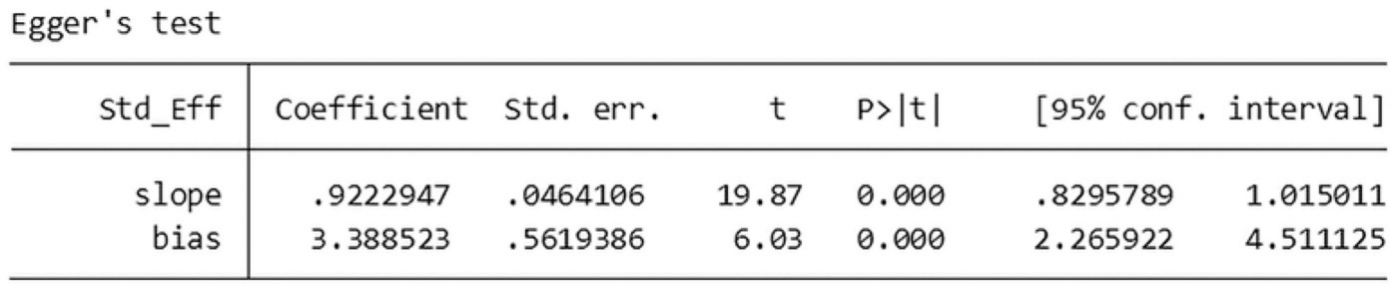
Egger's publication bias table.

Sensitivity analysis showed that the heterogeneity of the included studies may come from 6 studies (41, 47, 50, 62, 70, 91). Using the random effect model, when the 6 studies of each study were removed one by one, the heterogeneity was reduced to 25% (*P* = 0.04, *P* < 0.05), indicating that the heterogeneity of the total effective rate of the included studies was small and the stability was the best. In applying the random effect model, the sequential removal of each of the 6 studies resulted in a decrease in heterogeneity to 25% (*P* = 0.04, *P* < 0.05), suggesting that the included studies exhibited minimal heterogeneity and robust stability. The results of 66 studies (6, 9, 35, 36, 51, 52, 70, 71)^,^ (7, 9, 12, 13, 19, 21, 24, 44, 45, 49, 53, 56, 59, 64, 77, 78, 85–87) were stable. The results of Meta-analysis showed that the total effective rate of the experimental group was higher than that of the conventional treatment group, and the difference was statistically significant [RR = 1.22, 95%CI (1.19, 1.25), *P* < 0.05], suggesting that the experimental group had obvious advantages over the control group in the treatment of lumbar disc herniation. The results are shown in the forest plot of total effective rate comparison of the included studies in Figure 3, The forest plot in Figure 3 illustrates the comparative total effective rates of included studies, as detailed in the meta-analysis. as well as the funnel plot of total effective rate comparison of the included studies in Figure 4. The funnel plot in Figure 4 illustrates the comparison of total effective rates among the included studies, which is a critical tool for evaluating publication bias and the robustness of the meta-analysis. Egger's *P* < 0.0001, indicating a significant publication bias (Figure 8 Egger's publication bias plot and Figure 9 Egger's publication bias table).

### 4.2 Secondary outcome

#### 4.2.1 VAS score

In 48 studies (10–37, 40, 43, 46–58, 63–65, 67, 68, 73, 75, 77, 79, 83, 87, 88, 90), VAS was used to evaluate the pain relief of patients, and the lower the score, the more obvious the pain relief. In a study involving 5,770 patients, 2,879 were assigned to the experimental group and 2,891 to the control group, mirroring the common practice in medical research to have comparable numbers in each group to ensure statistical validity. Because the VAS scores of the included studies were different at different times, a meta-analysis was performed on the VAS scores of the included studies at different times.

Among them, 12 included studies (8, 25, 53, 58, 65, 68, 70, 75, 80, 83, 87, 90) used fixed effect model to conduct meta-analysis on VAS score at 1 day after surgery. A meta-analysis of 15 included studies (8, 11, 27, 29, 36, 43, 53, 65, 73, 75, 80, 83, 87, 88, 90) was performed on VAS scores at 1 week after surgery using a fixed effect model. 27 included studies (9, 10, 12, 13, 25, 27, 29, 31, 32, 43, 47, 49, 51, 53, 54, 65, 70, 73, 75, 79, 84, 88, 93) used fixed effect model to conduct meta-analysis on VAS score at 1 month after surgery. A meta-analysis of 30 included studies (9, 11, 25, 27, 29, 31, 32, 34, 37, 43, 46, 48–50, 58, 63, 64, 67, 77, 79, 84, 85, 88, 90) was performed on VAS scores at 3 months after surgery using a fixed effect model. 20 included studies (8, 14, 25, 27–29, 31, 40, 45, 47, 58, 67, 73, 77, 79, 85, 90) used fixed effects model to perform meta-analysis on VAS scores at 6 months after surgery. However, all the above studies showed high heterogeneity in the meta-analysis results, so the randomized effect model was used for the meta-analysis of the above-mentioned included studies. Among them, 5 studies (29, 53, 65, 73, 87) related to VAS score at 1 week after surgery were excluded, and the randomized effect model was used for the Meta-analysis. The heterogeneity of relevant studies is relatively large, which does not exclude the problem of incomplete relevant data caused by the low quality of the included literature. However, the differences between the studies were statistically significant, suggesting that the experimental group was However, the statistically significant differences observed between the studies imply that the experimental group exhibited a notable effect. The results are detailed in Table 3. Results of meta-analysis of VAS scores for secondary outcome measures. The Egger values obtained at one day, one week, one month, three months and six months after surgery were all greater than 0.05, indicating that there was no significant publication bias. The detailed outcomes of the meta-analysis on VAS scores for secondary outcome measures are presented in Table 3 (VAS score Supplementary Figure, S2–S21).

**Table 3:** The results of the meta-analysis of the visual analogue scale (VAS) scores for secondary indicators.

VAS	Literature quantity	Heterogeneity		Meta analysis model	MD（95%CI）	*P* value
		*P* value	*I* ^2^			
1 day	12	<0.00001	97%	Randomization	−0.49 [−1.01, 0.04]	0.07
1 week	10	0.03	52%		−0.70 [−0.91, 0.49]	<0.00001
1 month	27	<0.00001	98%		−1.40 [−1.72, −1.08]	<0.00001
3 month	30	<0.00001	97%		−1.15 [−1.38, 0.91]	<0.00001
6 month	20	<0.00001	99%		−0.83 [−1.29, −0.37]	0.0004

VAS, visual analogue scale.

#### 4.2.2 JOA score

In 10 studies (13, 15, 16, 25, 26, 29, 32, 46, 48, 88), JOA score was used to evaluate the lumbar spine dysfunction of patients, and the lower the JOA score, the more obvious the dysfunction was. A total of 884 patients were enrolled, including 442 in the experimental group and 442 in the control group. Because the VAS scores of the included studies were different at different times, a meta-analysis was performed on the JOA scores of the included studies at different times.

Among them, 3 included studies (13, 46, 48) used fixed effects model to analyze the JOA score at 2 weeks after surgery, 4 included studies (13, 25, 29, 88) used fixed effects model to analyze the JOA score at 1 month after surgery, and 7 included studies (13, 25, 29, 32, 46, 48, 88) used fixed effects model to analyze the JOA score at 3 months after surgery. However, all the above studies showed high heterogeneity in the meta-analysis results, so the random effects model was used for the meta-analysis of the above included studies. The findings indicate that despite the studies' efforts to standardize post-operative care, the variability in patient outcomes remains significant, suggesting that the heterogeneity among the studies persists. However, the differences in each study were statistically significant, suggesting that the experimental group had an effect in reducing postoperative lumbar spine dysfunction.

Among them, 5 included studies (15, 16, 25, 26, 29) used fixed effect model to conduct meta-analysis on JOA score at 6 months after surgery, and the results showed that *P* = 0.001, *I*^2^  =  78%. The statistical heterogeneity among the studies was large, and the random effect model was used for analysis. Due to the high statistical heterogeneity among the studies, the random effect model was adopted for further analysis. After excluding 1 study (29) one by one, the heterogeneity between the studies was reduced to 25%. The difference was statistically significant [MD = 3.50, 95%CI (2.65, 4.35), *P* < 0.00001]. Compared with the control group, the experimental group has obvious advantages in reducing lumbar spine dysfunction at 6 months after operation. The Egger values at two weeks, one month, three months and six months post-operation were all greater than 0.05, indicating that there was no obvious publication bias. The results are detailed in Table 4 Results of meta-analysis of JOA scores for secondary outcome measures (JOA score Supplementary Figures S22–S37).

**Table 4:** This is the results of meta-analysis of JOA scores for secondary outcome measures.

JOA	Literature quantity	Heterogeneity		Meta analysis model	MD（95%CI）	*P* value
		*P* value	*I* ^2^			
2 weeks	3	<0.00001	91%	Randomization	3.04 [0.44, 5.65]	0.02
1month	4	<0.00001	92%		4.46 [2.59, 6.34]	<0.00001
3months	7	<0.00001	95%		4.09 [2.36, 5.82]	<0.00001
6months	5	0.26	25%		3.50 [2.65, 4.35]	<0.00001

JOA, Japanese orthopaedic association assessment method.

#### 4.2.3 ODI score

In 10 studies (16, 26, 28, 29, 32, 45, 46, 70, 73, 93), ODI scores were used to assess the scale of the degree of functional impairment of patients in daily life, with lower ODI scores indicating less functional impairment of patients in daily life. A total of 952 patients were enrolled, including 477 in the experimental group and 475 in the control group. Because the ODI scores varied across the included studies at different time points, a meta-analysis was conducted on these scores.

Among them, 2 studies (46, 93) used fixed-effect model to analyze ODI scores at 2 weeks after surgery, and 5 studies (29, 45, 70, 73, 93) used fixed-effect model to analyze ODI scores at 1 month after surgery, 4 included studies (13, 29, 32, 93) used fixed effect model to conduct meta-analysis on ODI scores at 3 months after surgery, and 2 included studies (70, 93) used fixed effect model to conduct meta-analysis on ODI scores at 12 months after surgery. The heterogeneity among the studies was relatively small, and the differences were statistically significant. Studies suggest that patients in the experimental group, who underwent targeted interventions, exhibited greater improvements in reducing daily life dysfunction compared to those in the control group (Table 5).

**Table 5:** The results of the meta-analysis on the secondary indicator ODI score.

ODI	Literature quantity	Heterogeneity		Meta analysis model	MD（95%CI）	*P* value
		*P* value	*I* ^2^			
2weeks	2	0.18	44%	Fixation	−8.13 [−10.40, −-5.85]	<0.00001
1months	5	0.40	1%	Fixation	−6.59 [−7.84, −5.34]	<0.00001
3months	4	0.30	19%	Fixation	−8.06− [−9.34, −6.78]	<0.00001
6months	8	0.08	95%	Randomization	−7.63 [−9.21, −6.05]	<0.00001
12months	2	0.58	0%	Fixation	−4.90 [−7.42, −2.38]	0.0001

ODI, Oswestry disability index.

Among them, 8 studies (16, 26, 28, 29, 45, 70, 73, 93) were included in the meta-analysis of ODI scores at 6 months after surgery using the fixed effect model, and the results showed that *P* < 0.00001, *I*^2^  =  95%. The statistical heterogeneity between the studies was large, and the random effect model was used for analysis. After excluding 2 studies (16, 26), *I*^2^  =  48%, the heterogeneity between studies decreased, suggesting that the heterogeneity was still small, and the difference was statistically significant [MD = −7.63, 95%CI (−9.21, −6.05), *P* = 0.08]. Due to the small number of involved literatures at two weeks and twelve months after surgery, Egger values were not obtained. The Egger values at one month and three months after surgery were approximately 0.05, indicating no obvious publication bias. The Egger value at six months after surgery was less than 0.05, suggesting obvious publication bias. The results suggested that the experimental group was significantly better than the control group in reducing the dysfunction of daily life at 6 months after operation (ODI score Supplementary Figures S38–S49).

### 4.4 Safety evaluation

In into the 89 study, there are 13 (7, 9, 14, 28, 32, 53, 55, 57, 62, 66, 83, 91) research reported adverse events happening, has reported a total of 184 cases of adverse events, including experimental group 87 cases, control group of 97 cases in 1,712 cases, 856 cases of test group, There were 856 cases in the control group. The results showed that the incidence of adverse events in the experimental group and the control group was relatively uniform, and the difference was not statistically significant *P* > 0.05). Among them, the heterogeneity of postoperative lower limb pain and urinary retention in the adverse events was large, which could not be excluded because of the uneven quality of the included studies and the large degree of bias, resulting in a large difference in statistical analysis results. However, these two adverse events only involved 4 literature studies, which could not fully indicate the high incidence of these adverse events. The incidence of these two adverse events in the Meta-analysis was *P* > 0.05, so there was no statistical significance (AE Supplementary Figures S50–S80; Table 6).

**Table 6:** The comprehensive analysis results of the relevant literature's safety indicators.

Adverse effect	Literature quantity	Heterogeneity	RR (95%CI)	*P* value
Nausea, Vomiting	6 (9, 32, 51, 57, 62, 91)	0	1.70 [0.83, 3.47]	0.15
Headache	4 (32, 51, 55, 62)	0	1.30 [0.58, 2.89]	0.52
Numbness of limb	2 (14, 28)	0	2.00 [0.51, 7.77]	0.32
Postoperative leg pain was aggravated	2 (55, 62)	major	2.50 [0.49, 12.73]	0.27
cauda equina injury	2 (14, 28)	0	1.00 [0.21, 4.83]	1.00
Postoperative infection	2v (53, 57)	0	0.43 [0.17, 1.09]	0.08
Uroschesis	2 (7, 62)	major	0.67[0.24, 1.86]	0.44
Others	2 (53, 83)	0	1.00 [0.33, 3.05]	1.00
Dizzy giddy	1 (9)		1.00 [0.15, 6.79]	1.00
Psoas muscle Spasms	1 (91)		1.00 [0.26, 3.78]	1.00
Osphyalgia	1 (66)		1.25 [0.36, 4.32]	0.72
The pain gets worse	1 (91)		1.00 [0.35, 2.89]	1.00
Nerve root injury	1 (57)		0.55 [0.21, 1.41]	0.21
Disturbance of Sensation	1 (53)		0.20 [0.02, 1.68]	0.14
Acroparalysis	1 (53)		0.17 [0.02, 1.36]	0.09
Stand out again	1 (53)		0.33 [0.04, 3.15]	0.34

## 5 Discussion

Lumbar disc herniation (LDH) is a prevalent condition, primarily affecting middle-aged and elderly individuals. Recently, its incidence has shifted towards younger age groups, diversifying the patient population and significantly impacting individuals' quality of life and physical and mental wellbeing. LDH is a progressive condition with non-prominent early symptoms, often leading patients to opt for conservative treatment over an extended period. However, in daily life, patients have low compliance with conservative treatment, and often ignore the correction of bad habits in daily life, which will often lead to further development of the disease.

In this paper, a meta-analysis of 89 included studies was conducted to evaluate the efficacy and safety of radiofrequency thermocoagulation combined with conventional therapy. Clinical trial results show that the total effective rate of the group receiving radiofrequency thermocoagulation combined with conventional treatment is significantly higher than that of the conventional treatment group alone. When analyzing the VAS score and JOA score of the included studies, it was found that the heterogeneity among the studies was relatively large. Upon meta-analysis of the included studies, it was observed that the VAS and JOA scores, which are commonly used to assess treatment outcomes for spinal conditions, exhibited significant heterogeneity among the studies. After eliminating the literature one by one, the heterogeneity among the studies was not eliminated, indicating that the heterogeneity was still large, but the differences among the studies were statistically significant. Despite the sequential exclusion of studies, the heterogeneity persisted, indicating that the differences among the studies were statistically significant and not attributable to any single study. Therefore, it is suggested that radiofrequency thermocoagulation combined with conventional therapy is more effective in relieving postoperative pain than conventional therapy alone. In the analysis of ODI scores of the included studies, At six months postsurgery, a high heterogeneity was observed among the studies; however, upon excluding relevant studies, the heterogeneity decreased, indicating that the experimental group significantly outperformed the control group in alleviating patients' daily life dysfunction. Other studies have also shown that the experimental group is significantly better than the control group in reducing the disability of patients in daily life. In the safety evaluation of the included studies, it was found that the incidence of adverse events in the experimental group and the control group was relatively uniform, and most adverse events were usually relieved without intervention, so there was no statistical significance, suggesting that radiofrequency thermocoagulation target therapy combined with conventional treatment is safe, but further research is still needed.

Limitations of this study include:

1. There are differences in baseline characteristics and follow-up time of patients in many included studies, which may affect the accuracy of the results;
2. Many included studies did not explicitly allocate concealment and blinding methods, so there was a lack of high-quality studies;
3. There are many kinds of conventional treatments included in the study, and there may be large errors in the collection of efficacy data and adverse event data;
4. Although the sample size of this study was large, the time span of the included research was also long, and the evaluation criteria for efficacy also changed, these factors may have a relatively minor impact on the research results;
5. A small number of the included studies had differences in their diagnostic criteria, the time of collecting outcome indicators, and the types of outcome indicators. These differences might affect the final results of the meta-analysis;
6. The follow-up time mentioned in the included studies was short, and it was not possible to evaluate the postoperative efficacy for a long time. The follow-up duration noted in the studies was insufficient, hindering long-term assessment of postoperative efficacy.
7. The issue of language limitations: Most of the literatures included in this study were published in Chinese. Although professional English translation has been used to ensure the accuracy of data extraction, this may still create barriers to information access for non-Chinese readers: non-Chinese readers cannot directly access the original literatures to verify details such as research design, implementation standards of intervention measures, and measurement methods of outcome indicators. Consequently, this has a certain impact on the judgment of the transparency and reproducibility of the conclusions of this study.

In conclusion, studies have shown that radiofrequency thermocoagulation targeting, when combined with conventional treatments, significantly enhances the total effective rate for treating lumbar disc herniation. This approach is particularly effective in alleviating postoperative pain and diminishing lumbar spine dysfunction, while maintaining a low rate of adverse events and ensuring a high level of safety. However, this study still has some limitations. In response to the language limitation in this study, future research may consider collaborating with Chinese-English research teams to expand the language scope of included literatures, thereby enhancing the generalizability of evidence. Additionally, supplementary English explanations for core methodological information of key Chinese studies (such as research protocols and summaries of raw data) should be provided to improve the international accessibility of evidence. The quality of the included studies is poor, potentially leading to inaccuracies or missing data, thereby affecting the credibility. Therefore, more scientific, multi-center, large-sample and high-quality clinical trials are still needed to verify the above conclusions, so as to provide reliable evidence for clinical decision-making.

## Data Availability

The original contributions presented in the study are included in the article/Supplementary Material, further inquiries can be directed to the corresponding author.
